# Kidney-based in vivo model for drug-induced nephrotoxicity testing

**DOI:** 10.1038/s41598-020-70502-3

**Published:** 2020-08-14

**Authors:** Yuan-Yow Chiou, Si-Tse Jiang, Yu-Sian Ding, Yu-Hsuan Cheng

**Affiliations:** 1 Institute of Clinical Medicine, Medical College, National Cheng Kung University, Tainan, 70403 Taiwan; 2 Division of Pediatric Nephrology, Department of Pediatrics, National Cheng Kung University and Hospital, 138 Sheng-Li Road, Tainan, 70403 Taiwan; 3 National Laboratory Animal Center, National Applied Research Laboratories, Tainan, 74147 Taiwan

**Keywords:** Kidney, Kidney

## Abstract

The need is critical and urgent for a real-time, highly specific, and sensitive acute kidney injury biomarker. This study sought to establish a sensitive and specific *Miox-NanoLuc* transgenic mouse for early detection of drug-induced nephrotoxicity. We generated *Miox-NanoLuc* transgenic mice with kidney-specific NanoLuc overexpression. Our data showed that Miox-NanoLuc-produced luminescence was kidney-specific and had good stability at room temperature, 4 °C, − 20 °C, and repeated freeze–thaw cycles. Serum levels of BUN and creatinine were significantly increased at day 2 or 3 in cisplatin-treated mice and at day 5 in aristolochic acid (AAI)-treated mice. Particularly, the serum and urine Miox-NanoLuc luminescence levels were significantly increased at day 1 in cisplatin-treated mice and at day 3 in AAI-treated mice. Renal pathological analysis showed that the kidney sections of cisplatin-treated mice at day 5 and AAI-treated mice at day 13 showed cytolysis and marked vacuolization of tubular cells. In conclusion, we developed a new platform to early quantify drug-induced nephrotoxicity before serum BUN and creatinine levels increased and pathological tubular cell injury occurred. This model may serve as an early detection for drug- and food-induced nephrotoxicity and as an animal model to investigate tubular cell injury.

## Introduction

Chronic kidney disease is a type of kidney disease that affects 8–16% of population worldwide^1^. It is characterized by gradual loss of key function over time, which eventually leads to kidney failure and requires dialysis or a kidney transplantation to maintain life^2^. Moreover, recent studies further revealed that acute kidney injury (AKI) positively associated with the risk of chronic kidney disease, and patients with chronic kidney disease complicated with AKI have a higher mortality rate^3–5^. Even with advancement of medical technology, the incidence of AKI has gradually increased the recent years and is associated with an increased risk of mortality^6–8^. Multiple risk factors might contribute to the occurrence of AKI, including food agents (aristolochic acid, mushrooms, dietary supplements, melamine, medicinal traditional herbals), drugs, infection, ischemia, sepsis, and intravenous contrast^2,9–12^. In particular, drug-induced nephrotoxicity is a common factor accounting for approximately 60% of AKI cases in in hospitalized patients^13,14^. Despite efforts have been made to discovery drugs for the treatment of AKI^15^, no effective therapeutic candidates have been identified in the clinic.

Since there is a direct correlation between the detection time of kidney failure and mortality, early understanding of the characteristics of drugs that may cause AKI and timely intervention can reduce the morbidity and mortality^16^. In current clinical practice, AKI is usually diagnosed by measuring urine and serum biomarkers, including creatinine, blood urea nitrogen (BUN), interleukin-18 (IL-18), kidney injury molecule-1 (KIM-1), and neutrophil galatinase-associated lipocalin (NGAL)^16–19^. Unfortunately, these biomarkers are unreliable indicators during acute changes in kidney function^16^. Before observing the changes in the values of these biomarkers, the glomerular filtration rate (GFR), which reflects renal function, has been greatly reduced^20,21^. Therefore, there is an urgent need for highly specific and sensitive AKI biomarkers, especially for AKI drug screening platforms.

Myo-inositol oxygenase (Miox) is the rate-limiting enzyme of inositol catabolism that catalyze the oxidative cleavage of myo-inositol to produce d-glucuronic acid^22,23^. Urinary myo-inositol and Miox tissue expression were associated with the initial estimated GFR in focal segmental glomerulosclerosis patients^24^. Recent studies further found that Miox is a kidney-specific proximal tubule protein that increased in the serum of animal with AKI or diabetic nephropathy and the plasma of patients with severe AKI patients or type 2 diabetes mellitus^25–27^. In addition, cadmium-induced toxicity in mice kidney significantly increased Miox levels in the tubular cells^28^, and the Miox concentration in the serum and urine may negatively reflect the rental function^29^. Since Miox is a highly conserved enzyme in mouse, rat, and human kidney, Miox is likely to be an ideal biomarker of AKI for early detection of kidney injury. Since NanoLuc luciferase (Nluc) has higher sensitivity than the commonly used bioluminescent firefly luciferase (FFluc)^30,31^, the purpose of this study was to develop a new *Miox-NanoLuc* transgenic mice platform to quantify the drug-induced nephrotoxicity by detecting the kidney-specific Miox-NanoLuc produced luminescence in mouse serum and urine.

## Materials and methods

### Animals

Wild-type (WT) and *Miox-NanoLuc* transgenic mice were maintained at the National Laboratory Animal Center, National Applied Research Laboratories (Tainan, Taiwan) following the guidelines of the Care and Use of Laboratory Animals (National Institutes of Health). All animal experiments used in this study were approved by the Institutional Animal Care and Use Committee (IACUC) at National Laboratory Animal Center (No. NLAC (TN)-106-M-022).

### Generation of *Miox-NanoLuc* transgenic mice

**Figure 1:**
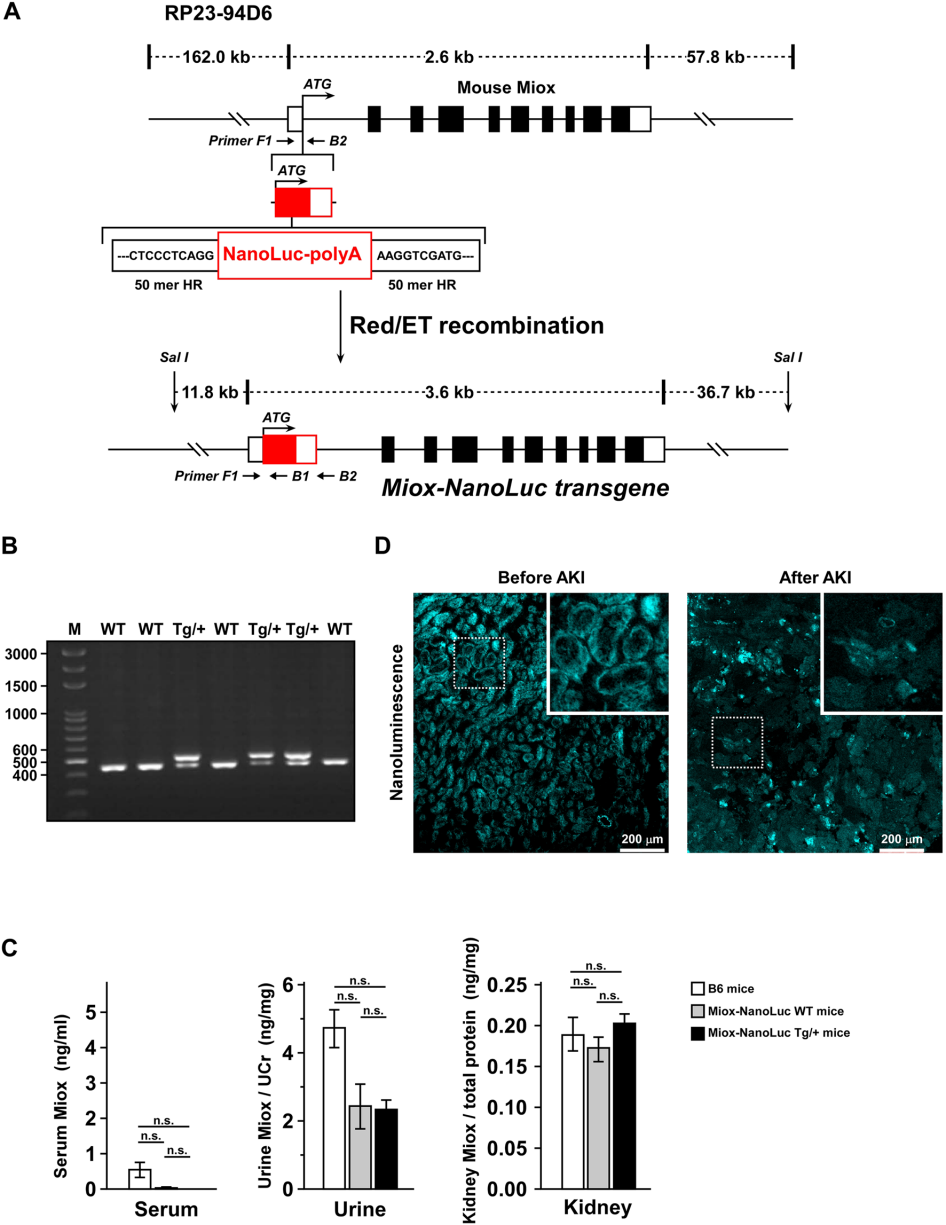
Characterization of *Miox-NanoLuc* mice. (**A**) Schematic presentation of the construction process of transgenic *Miox-NanoLuc* mice. The transgene was constructed using the mouse BAC clone RP23-94D6 as backbone with an inserted *Miox* gene containing its 5′UTR and 3′UTR. The NanoLuc-PolyA expression cassette was inserted into the start codon of *Miox* by RED/ET recombination system as described in “Materials and methods”. (**B**) PCR genotyping of wild type and transgenic *Miox-NanoLuc* mice. RT–PCR was performed to examine the wild-type (WT), heterozygous *Miox-NanoLuc* mice (Tg/+) using different primer sets as described in “Materials and Methods”. A single PCR product of 449-bp in length was amplified from WT mice, while the two 449-bp and 514-bp PCR fragments were amplified from the Tg/+ mice. (**C**) The expression of Miox protein in serum, urine and kidney were further confirmed by ELISA. There were no significant differences in the Miox expression between B6 mice, WT, and Tg/+ transgenic mice. Quantitative data were represented as mean ± standard error (SE). The n.s. symbol indicated no significance statistically. (**D**) Localization of Miox-NanoLuc in the proximal renal tubule cells. Before and after cisplatin treatment, the localization of Miox-NanoLuc luminescence signal in the kidney was determined by histological analysis.

A mouse bacterial artificial chromosome (BAC) clone RP23-94D6 covering the whole *Miox* gene was obtained from BACPAC Resources Center at the Children’s Hospital of Oakland Research Institute. The *NanoLuc-PolyA* expression cassette was inserted into the start codon of *Miox* in the BAC clone using the RED/ET recombination technique (Gene Bridges, Heidelberg, Germany). Briefly, the 50 mer homologous arms (HR, black capital letters in Fig. 1A) flanking the start codon on the exon 1 was capped upon a counter selector *rpsL-neo* by polymerase chain reaction (PCR), and was electroporated into *E. coli.* hosting the BAC clone to insert the *rpsL-neo* by homologous recombination. The *NanoLuc-polyA* (Red capital letters in Fig. 1A) capping the same 50 mer homologous arms was then used to replace the counter selector *rpsL-neo* again by homologous recombination so as to construct the transgene *Miox-NanoLuc* (Fig. 1A). The detailed protocol described in the instruction manual of the counter selection BAC modification kit (Gene Bridges, Heidelberg, Germany). The BAC transgene was purified, Sal I digest, and pulsed-field gel electrophoresis to isolate the 52.1 kb transgene for C57BL/6 mouse pronuclear microinjection.

### PCR genotyping of wild type and transgenic *Miox-NanoLuc* mice

Cut off the toes of the mice and added 200–300 µl of Direct PCR Lysis Reagent (Viagen Biotech, CA) containing freshly prepared 0.2–0.4 mg/ml Proteinase K (Sigma, MO), and rotated the tubes in rotating hybridization oven at 55 °C for 5 h or until no tissue clumps. The tubes were then incubated at 85 °C for 45 min. After incubation, the genomic DNA was stored at − 20 °C for genotyping analysis. Genotyping of the mice was performed by PCR analysis using above genomic DNA samples. The primers used in this PCR genotyping included Primer F1: 5′-CATTAACTTGCTGAGGTCAGGAGG-3′, Reverse B1: 5′-CGTCCGAAATAGTCGATCATGTTC-3′, and Reverse B2: 5′-TTAAGAGGCAGTGATCTCCACCTG-3′. For wild-type mice, a single PCR product of 449-bp in length was amplified. For the heterozygous transgenic mice (Tg/+), the two 449-bp and 514-bp PCR fragments were amplified. The PCR conditions used in this study were as follows: pre-denaturation at 95 °C for 2 min, followed by 35 amplification cycles of denaturation at 95 °C for 30 s, primer annealing at 60 °C for 30 s, and extension at 72 °C for 45 s, and finally an additional extension at 72 °C for 10 min.

### Drug-induced nephrotoxicity

Eight-week-old male WT mice and age-matched *Miox-NanoLuc* transgenic male mice were used for examining the drug-induced nephrotoxicity. Various doses of cisplatin (10, 20, or 40 mg/kg body weight) or Aristolochic acid I (AAI, Sigma-Aldrich; 2 or 3.5 mg/kg body weight) were injected intraperitoneally in *Miox-NanoLuc* transgenic mice. The vehicle control mice were injected with normal saline (cisplatin group) or corn oil (AAI group). After collecting the blood and urine samples from each mouse, the levels of nephrotoxic biomarkers in the blood and urine were measured. The kidneys of the mice were used to examine various morphologic, biochemical, and pathologic studies.

### Measurement of the creatinine and BUN levels in serum and urine

After collecting blood from each mouse, the blood was allowed to stand at room temperature for 30 min, and then centrifuged at 6,000 rpm at 4 °C to collect upper serum. Serum creatinine and BUN levels were analyzed by FUJI DRI-CHEM 4000i analyzer (FUJIFILM Corp., Tokyo, Japan). On the other hand, urine creatinine level was detected by Creatinine Colorimetric Detection kit (Enzo Biochem, NY) according to the manufacturer protocol. Briefly, the mouse urine was diluted with deionized water to a final 50 µl reaction volume in a 96-well plate. For each well, 100 µl of Creatinine Detection Reagent was added and incubated for 30 min. Plate-based colorimetric measurement (490 nm) was performed by Epoch microplate spectrophotometer (Biotek, VT).

### In vivo imaging system (IVIS) assay

Bioluminescence image was detected using an IVIS Xenogen system (PerkinElmer, MA). The Nano-Glo Luciferase Assay Substrate (Promega, WI) was diluted 1:20 in PBS, and 0.1 ml of the solution was injected into the tail vein of the mouse. Mice were anesthetized with isoflurane during imaging. Image acquisition and analysis were performed using Living Image software (PerkinElmer, MA). Flux measurements were acquired from regions of interest, which were automatically gated to the signal contours.

### Measurement of luminescence intensity of serum, urine and tissue samples

The luminescence intensity in serum and urine was measured in a 96-well plate with a final reaction volume of 100 µl. Each well was then added 100 µl of Nano-Glo Luciferase Assay Reagent and mix for optimal consistency. The luminescence intensity was measured by Molecular Devices LMAX II 384 Microplate Reader (Molecular Devices, CA). In order to measure the luminescence intensity of mouse tissue, the incised mouse tissues of liver, heart, spleen, lung and kidney were treated with 0.5 µl of Reporter Lysis Buffer (Promega, WI), ground, and sonicated for 1 min. The sample was centrifuged at 4 °C for 20 min, and the supernatant was collected to analyze the protein concentration by Protein Assay Dye Reagent Concentrate kit (Bio-Red, CA). The following measurement steps were the same as above.

### Enzyme-linked immunosorbent assay (ELISA)

ELISA was used to detect the levels of urine KIM-1 (R&D system, MN), cystatin C (R&D system, MN), clusterin (R&D system, MN), β2-microglobulin (MyBioSource, CA), trefoil factor-3 (Cloud-clone corp., TX), and Miox (MyBioSource, CA). Briefly, 100 µl of Assay Diluent was added into 96-well microplate, and 50 µl of standard, control, or sample were then added to each well. After incubation at 4 °C for 2 h, each well was washed 3 times and added detection antibody at 4 °C for 2 h. After washed with PBS, HRP-conjugated antibody was added into each well and further incubated for 1 h. After PBS washing, TMB substrates were added to each well for 20 min, and stop solution was added to stop the reaction. The absorbance of each well was measured by Epoch microplate spectrophotometer (Biotek, VT).

### Hematoxylin and Eosin (H&E) staining

After sacrifice, the mouse kidney tissues were fixed with 10% formalin and embedded in paraffin. The embedded tissues were then sliced into 3 μm thick section, mounted on glass slides, and incubated at 75 °C for 30 min. After deparaffinization using xylene for 10 min, the specimens were rehydrated using a graded ethanol series (95%, 85%, and 70% ethanol). After washing, the specimens were treated with Hematoxylin for 2 min, washed in running tap water for 1 min, and then incubated with acid alcohol for 1 s. Afterwards, the specimens were incubated with ammonia water solution for 1 s and washed in running tap water for 10 min. After counterstaining in Eosin solution for 90 s, the specimens were dehydrated using 70%, 80%, 90%, and 100% ethanol. Finally, the specimens were mounted with mounting medium, and the kidney injury was observed under the microscopy.

### Statistical analysis

Statistical data were expressed as mean ± standard error (SE). Difference of the expression intensities between wild type mice and *Miox-NanoLuc* transgenic mice was compared using two-sample t-test. Difference in serum BUN, serum creatinine, serum luminescence, urine luminescence, urine luminescence/creatinine, and change of body weight after treating with vehicle, cisplatin (10, 20, or 40 mg/kg), or AAI (2 or 3.5 mg/kg) were compared using one-way ANOVA with a post hoc-pairwise, Dunnett’s test as comparing with vehicle-treated group for each follow-up time point. A non-parametric method, Kruskall–Wallis test with post hoc-pairwise, Mann–Whitney test when the baseline measurement (at Day 0) didn’t follow normal distribution. All data were graphed using GraphPad Prism 6 for Windows (version 6.01)^©^ 1992–2012 GraphPad Software, Inc. All statistical analyses were carried out with IBM SPSS statistical software version 22 for Windows (IBM, NY).

## Results

### Establishment of *Miox-NanoLuc* transgenic mice

According to analysis results using bioinformatic database (BioGPP, https://biogps.org; GTX, https://www.informatics.jax.org/expression.shtml; GENEvisible, https://genevisible.com/search), Miox was found to be specifically expressed in epithelium of proximal tubule. Therefore, Miox was selected as the host promoter for renal-specific transgene expression. The *NanoLuc-PolyA* expression cassette was inserted into the start codon of *Miox* gene in a BAC clone (RP23-94D6) by RED/ET recombination system, resulting in *NanoLuc-PolyA* instead of *Miox* expression driven by *Miox* promoter. The BAC transgene was purified, Sal I digested, and pulsed-field gel electrophoresis to isolate the 52.1 kb transgene for C57BL/6 mouse pronuclear microinjection (Fig. 1A). We got 26 pups in which two founders (line 6 and 28) carry the transgene, which was verified by genotyping PCR. As shown in Fig. 1B, a single PCR product of 449-bp in length was amplified in wild type mice, while, the two 449-bp and 514-bp PCR fragments were amplified in heterozygous transgenic mice (Tg/+). In addition, the expression levels of Miox in serum, urine, and kidney were determined by ELISA. There was no significant difference in Miox expression between B6 mice, wild-type mice and *Miox-NanoLuc* transgenic heterozygous mice (Fig. 1C). To further confirm whether Miox-NanoLuc was localized in the proximal tubular cells, histological analysis was performed to examine the luminescent NanoLuc signal in the kidney of NanoLuc transgenic mice. Strong luminescence intensity of NanoLuc was indeed localized in the renal tubule cells (Left panel, Fig. 1D). However, the luminescence intensity of NanoLuc in the renal tubule cells disappeared dramatically after cisplatin treatment of *Miox-NanoLuc* transgenic mice (Right panel in Fig. 1D). This result confirmed that Miox-NanoLuc was localized in proximal tubular cells, and release during AKI.

### Tissue-specific expression of Miox-NanoLuc in kidney

**Figure 2:**
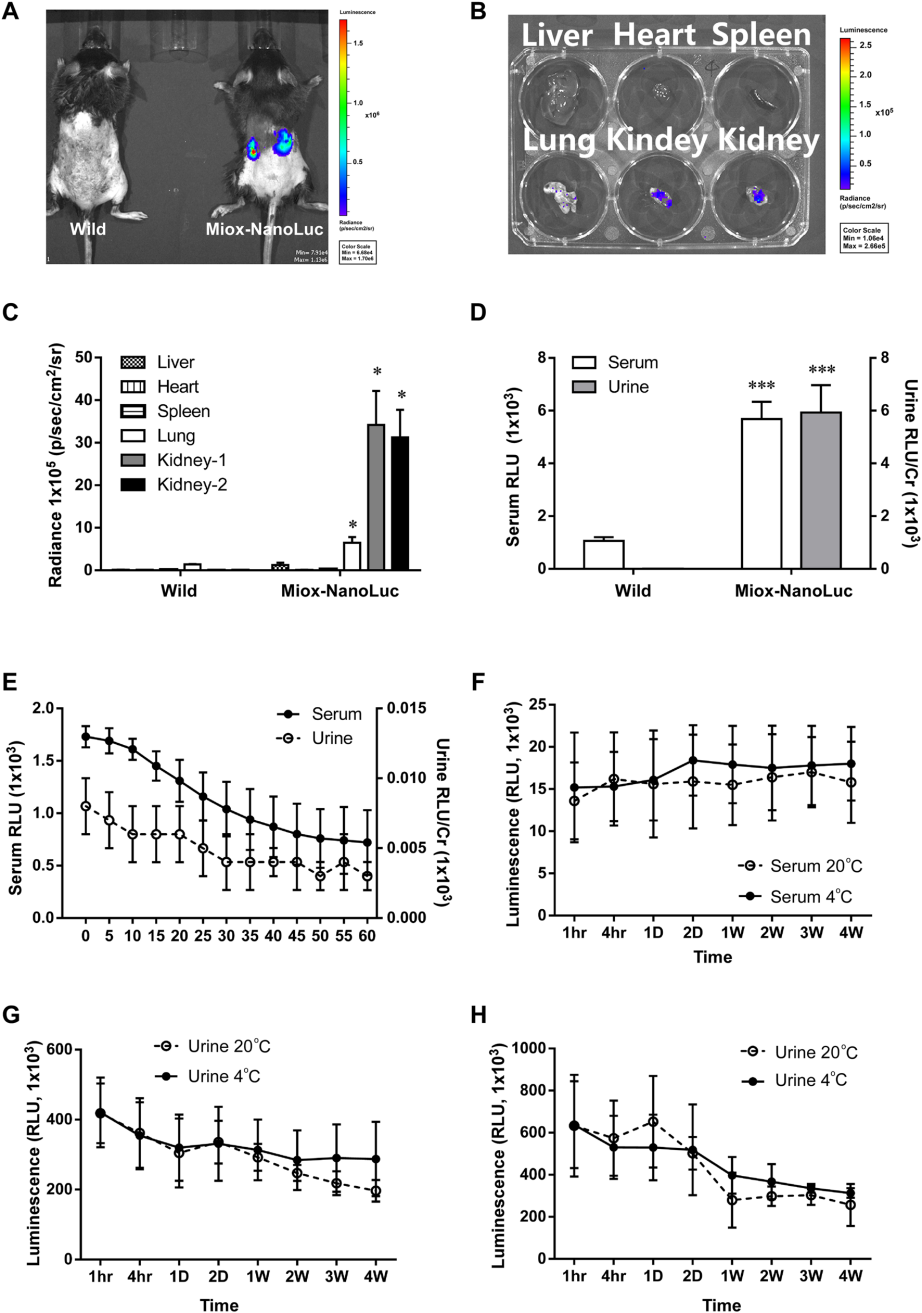
Tissue-specific expression of Miox-NanoLuc in mice and the stability of *Miox-NanoLuc* luminescence. (**A**) Representative in vivo bioluminescent imaging of the WT and *Miox-NanoLuc* mice using XENOGEN IVIS system. Blue represented lower radiance, and red represented higher radiance. (**B**) Luminescence intensity of Miox-NanoLuc in liver, heart, spleen, lung, and kidney. (**C**) Quantification of luminescence intensities of Miox-NanoLuc in major organs. Strong luminescence intensities observed in kidney organs. (**D**) *Miox-NanoLuc* luminescence intensity in serum and urine. Compared to WT mice, the *Miox-NanoLuc* transgenic mice has a strong Miox-NanoLuc luminescence intensity in serum and urine. (**E**) Stability of serum and urine Miox-NanoLuc in room temperature. Serum and urine Miox-NanoLuc luminescence intensities were measured every 5 min within 60 min at room temperature. (**F**) Stability of serum Miox-NanoLuc luminescence at 4 °C and − 20 °C. The serum was aliquoted and stored at 4 °C or − 20 °C for 4 weeks, and the luminescence intensity of serum at various time points was measured. (**G**) Stability of urine Miox-NanoLuc luminescence at 4 °C and − 20 °C. The urine was aliquoted and stored at 4 °C or − 20 °C for 4 weeks, and the luminescence intensity of urine at various time points was measured. (**H**) Within 4 weeks storage, the urine was repeated freeze–thaw at various time points, and then the luminescence intensity was measured. Radiance (means ± SEM; n = 6 mice). *< 0.05, **p < 0.01, and ***p < 0.001 were considered statistically significance between *Miox-NanoLuc* transgenic mice and WT mice.

The expression of Miox-NanoLuc in *Miox-NanoLuc* transgenic mice was further confirmed by IVIS analysis system. A shown in Fig. 2A, a strong luminescence intensity was observed only in the kidney of *Miox-NanoLuc* transgenic mice, but not in wild-type mice. Furthermore, Miox luminescence in liver, heart, spleen, lung and kidney tissues of Miox-NanoLuc transgenic mice was further examined. The luminescence intensity was significantly increased in the kidney of *Miox-NanoLuc* transgenic mice (Fig. 2B,C). Consistent result was also observed in various tissue of *Miox-NanoLuc* transgenic mice by luciferase activity assay (Supplementary Fig. 1). We then examined the Miox luminescence in the serum and urine of *Miox-NanoLuc* transgenic mice. Compared to wild-type mice, Miox-NanoLuc transgenic mice have significant elevated serum and urine luminescence levels (Fig. 2D). These results indicated that Miox-NanoLuc is specifically expressed in the serum, urine and kidney tissues of *Miox-NanoLuc* transgenic mice.

### Good stability of Miox-NanoLuc luminescence

To examine the stability of Miox-NanoLuc luminescence in serum and urine, the serum and urine of *Miox-NanoLuc* transgenic mice were stored at room temperature, 4 °C or − 20 °C. Afterwards, the luminescence intensity at each storage time point was further evaluated. As shown in Fig. 2E, luminescence intensity can still be detected in serum and urine after storing at room temperature for 60 min. The luminescence in serum and urine can be stably detected even if stored at 4 °C or − 20 °C for 4 weeks (Fig. 2F,G). In addition, the luminescence was still detected in serum and urine after repeated freeze–thaw (Fig. 2H).

### Miox-NanoLuc luminescence is as an early biomarker for drug-induced nephrotoxicity

**Figure 3:**
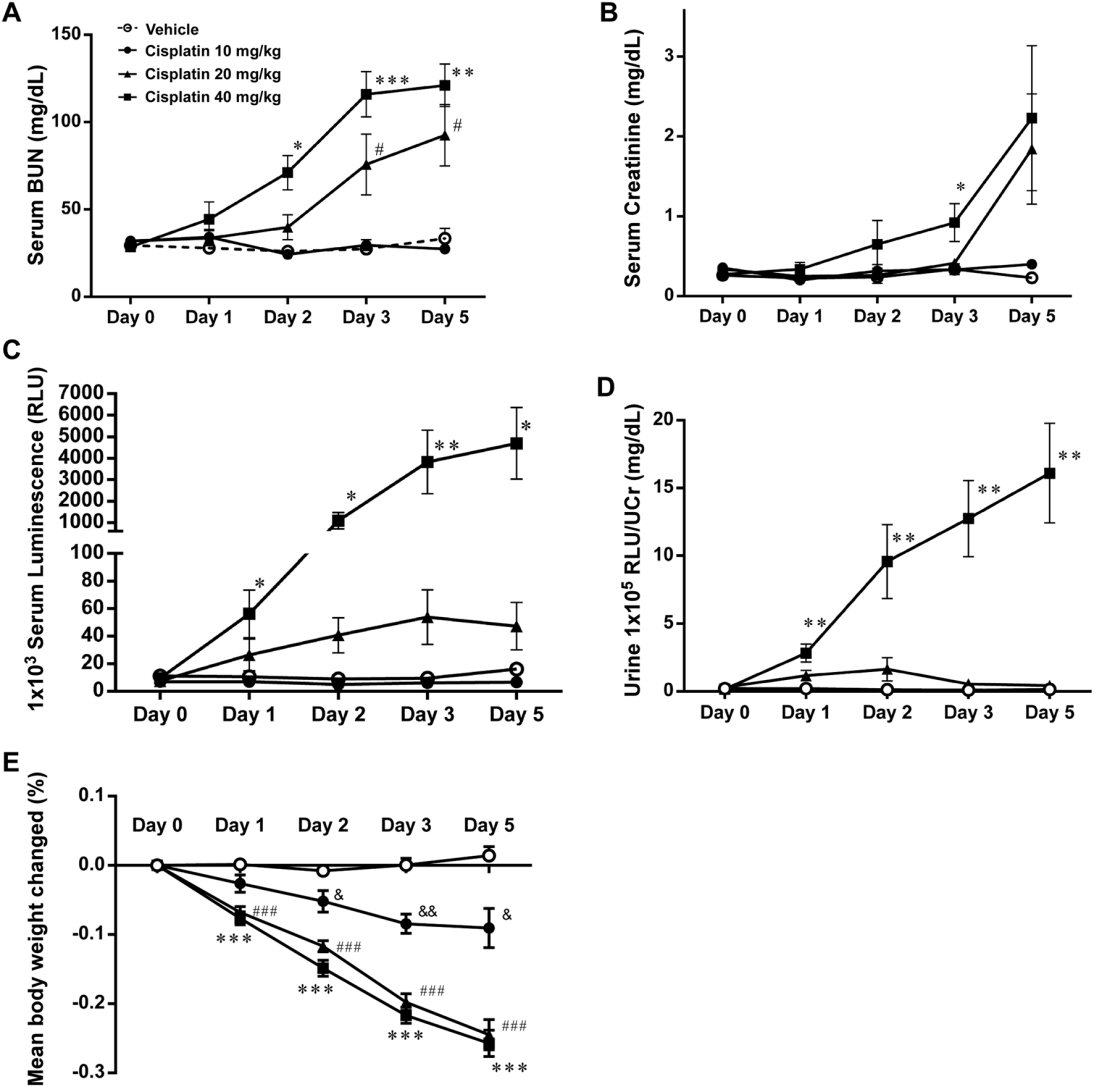

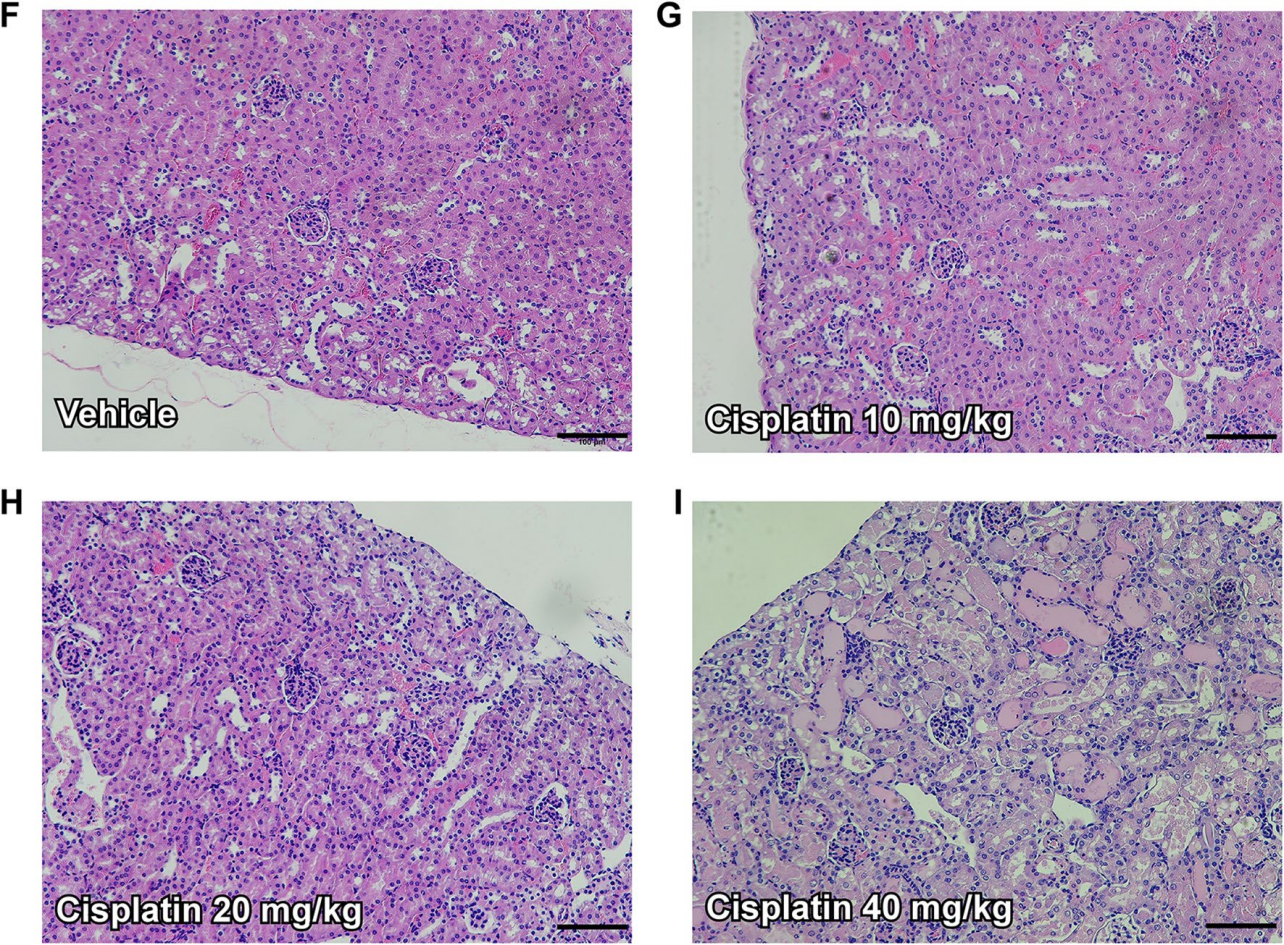
Miox-NanoLuc luminescence as an early biomarker for cisplatin-induced nephrotoxicity. After intraperitoneal injection of vehicle or cisplatin (10, 20, or 40 mg/kg), the levels of (**A**) serum BUN, (**B**) serum creatinine, (**C**) serum Miox-NanoLuc luminescence, (**D**) urine Miox-NanoLuc luminescence, and (**E**) percent of change in mouse body weight were measured at day 0, 1, 2, 3, and 5. The pathological renal damage caused by vehicle (**F**), 10 mg/kg cisplatin (**G**), 20 mg/kg cisplatin (**H**), and 40 mg/kg cisplatin were detected by H&E staining at day 5. Data were presented as mean ± SE and compared using one-way ANOVA with a post hoc-pairwise, Dunnett’s test. *< 0.05, **p < 0.01, and ***p < 0.001 were considered to be statistically significance between vehicle and 40 mg/kg cisplatin. # < 0.05, ##*p* < 0.01, and ###*p* < 0.001 were considered to be statistically significance between vehicle and 20 mg/kg cisplatin. & < 0.05 and &&* p* < 0.01 were considered to be statistically significance between vehicle and 10 mg/kg cisplatin.

**Figure 4:**
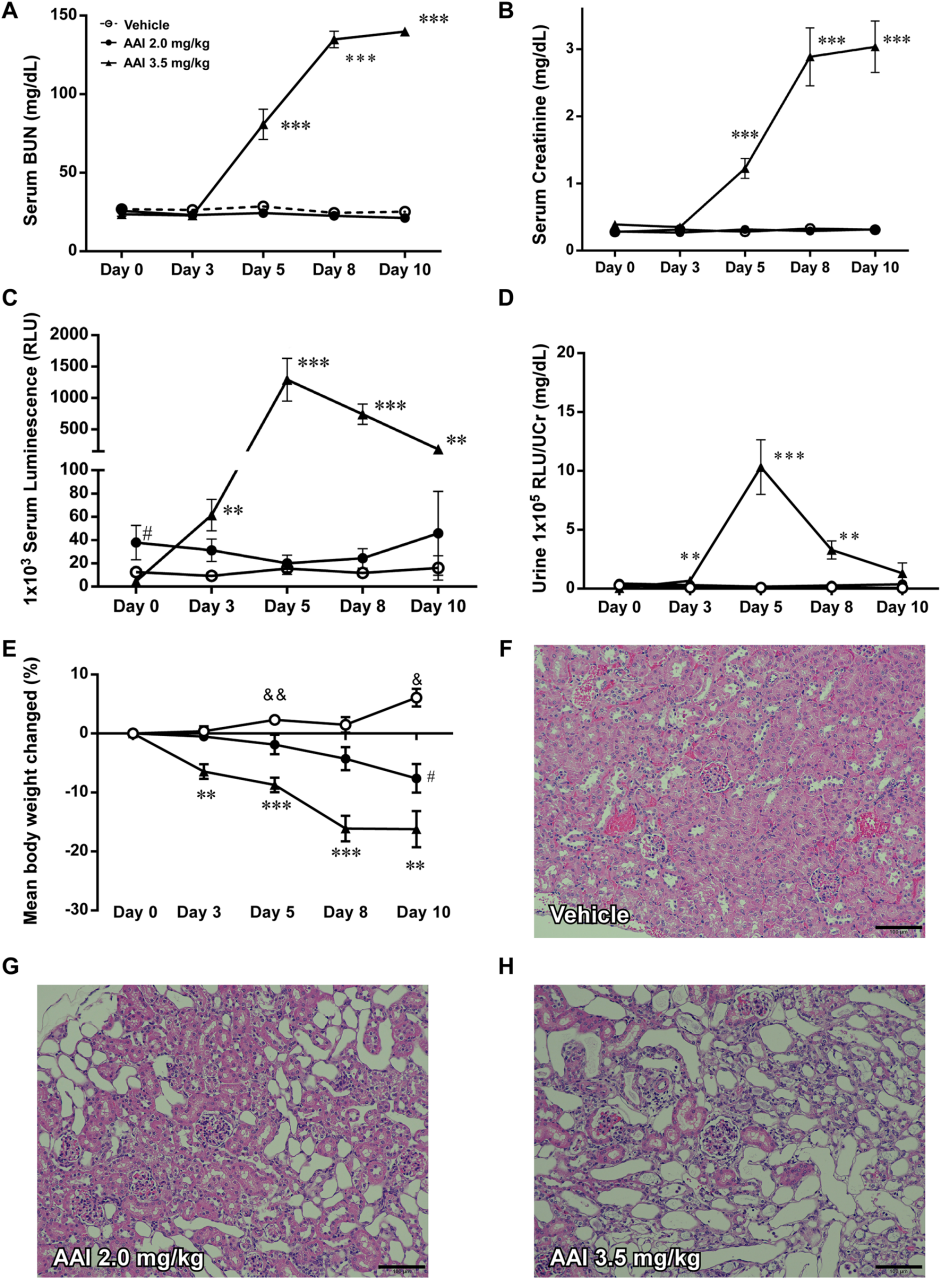
Miox-NanoLuc luminescence as an early biomarker for AAI-induced nephrotoxicity. After intraperitoneal injection with vehicle or aristolochic acid I (AAI) (2 or 3.5 mg/kg), the levels of (**A**) serum BUN, (**B**) serum creatinine, (**C**) serum luminescence, (**D**) urine luminescence, and (**E**) percent of change in mouse body weight were measured at day 0, 3, 5, 8 and 10. The pathological renal injury caused by vehicle (**F**), 2.0 mg/kg AAI (**G**), and 3.5 mg/kg AAI (**H**) were detected by H&E staining at day 13. Data were presented as mean ± SE and compared using one-way ANOVA with a post hoc-pairwise, Dunnett’s test. *p < 0.05, **p < 0.01, and ***p < 0.001 were considered to be statistically significance between vehicle and 3.5 mg/kg AAI. ^#^ < 0.05, ^##^*p* < 0.01, and ^###^*p* < 0.001 were considered to be statistically significance between vehicle and 2.0 mg/kg AAI.

Next, we examined whether Miox-NanoLuc luminescence can be detected earlier than BUN and creatinine under drug-induced nephrotoxicity. As shown in Fig. 3A,B, serum BUN and creatinine levels were significantly increased after 2 or 3 days of cisplatin treatment (40 mg/kg). Similarly, serum BUN and creatinine levels were also significantly increased after 5 days of AAI treatment (3.5 mg/kg; Fig. 4A,B). Remarkably, serum Miox-NanoLuc luminescence was significantly increased after 1 day of cisplatin treatment (Fig. 3C) and 3 days of AAI treatment (Fig. 4C). Consistently, urine Miox-NanoLuc luminescence increased significantly after 1 day of cisplatin treatment (Fig. 3D) and 3 day of AAI treatment (Fig. 4D), indicating that changes of Miox-NanoLuc can be detected earlier than creatinine and BUN. On the other hand, cisplatin (Fig. 3E**)** and AAI treatment (Fig. 4E) resulted in weight loss. Next, we determine whether renal tubular injury was occurred after cisplatin and AAI treatment. The glomeruli of vehicle-treated mice had no collapse of the capillary loops and pathological injury (Fig. 3F,F), while the renal tubular cells of cisplatin-treated mice (10 and 20 mg/kg) showed little renal tubular injury (Fig. 3G,H). However, in the 40 mg/kg cisplatin-treated mice, renal tubular cells showed cytolysis, detachment from the underlying basal lamina, and loss of brush border as well as tubular cell vacuolization (Fig. 3I). Similarly, AAI treatment also caused tubular cell lysis, detachment from the basal membrane, and vacuolization (Fig. 4G,H, and Supplementary Fig. 2). In summary, the above results indicated that Miox-NanoLuc luminescence can not only reflect cisplatin- and AAI-induced nephrotoxicity, but also can be detected before the pathological change of renal tubules damage.

**Figure 5:**
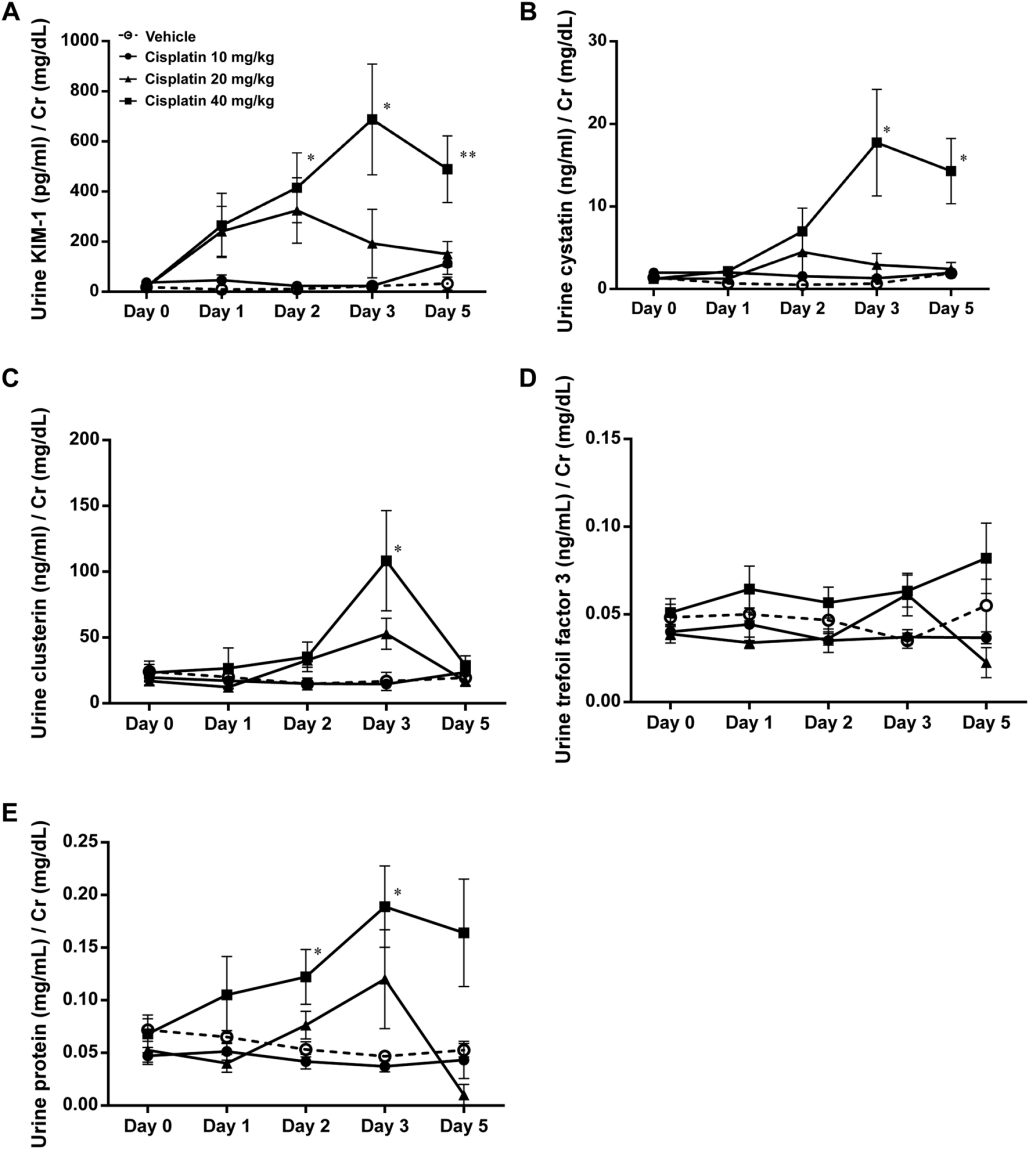
The ability of KIM-1, cystatin, clusterin, and trefoil factor 3 biomarkers in early detection of cisplatin-induced nephrotoxicity. After were intraperitoneal injection with vehicle or cisplatin (10, 20, or 40 mg/kg), the levels of (**A**) urine KIM-1, (**B**) urine cystatin C, (**C**) urine clusterin, (**D**) urine trefoil factor 3, and (**E**) urine protein were measured at day 0, 1, 2, 3, and 5. Data were presented as mean ± SE and compared using one-way ANOVA with a post hoc-pairwise, Dunnett’s test. *< 0.05, **p < 0.01, and ***p < 0.001 were considered to be statistically significance between vehicle and 40 mg/kg cisplatin. # < 0.05, ##*p* < 0.01, and ###*p* < 0.001 were considered to be statistically significance between vehicle and 20 mg/kg cisplatin. & < 0.05 and &&* p* < 0.01 were considered to be statistically significance between vehicle and 10 mg/kg cisplatin.

We further examined the abilities of other biomarkers such as KIM-1, cystatin, clusterin, and trefoil factor 3 in the early detection of drug-induced nephrotoxicity. As shown in Fig. 5, KIM-1, cystatin, clusterin, and urine proteins began to increase significantly after 2–3 days of 40 mg/ kg cisplatin treatment. However, trefoil factor 3 level was not changed during cisplatin treatment (Fig. 5D). Taken together, these results suggested that Miox-NanoLuc luminescence can be used as an early biomarker for drug-induced nephrotoxicity.

## Discussion

Since serum creatinine is insensitive and nonspecific for identifying kidney injury^21,32^, creatinine is an unreliable biomarker for acute changes in renal function. Before the level of creatinine increased, the glomerular filtration rate (GFR) may be significantly reduced. Up to 50% of renal function may have already been lost before creatinine may change. Consequently, creatinine is a poor biomarker for AKI due to its inability to help diagnose early acute kidney failure or differentiate among its various causes^16^. The present study further showed that, compared to creatinine and BUN levels, the Miox-NanoLuc intensity was significantly increased at the early stage of cisplatin- or AAI-induced AKI. In particular, a significant change in serum luminescence appeared in drug-treated mice before pathological changes occurred, suggesting that the Miox is an suitable biomarker that reflects drug-induced nephrotoxicity before the pathological changes.

Although urinary and serum proteins have been intensively investigated as potential biomarkers for the diagnosis of kidney injury, there is still a lack of ideal biomarkers for AKI. All of the potential biomarkers identified so far have their own strengths and weaknesses, so that a combination of biomarkers might be the optimal solution. In the present study, urine luminescence significantly increased in cisplatin-treated *Miox-NanoLuc* transgenic mice on day 1, but the levels of KIM-1, clusterin, cystatin, trefoil factor 3, and urine protein were not significant increased. The results were also supported by Vinken et al.^33^ that KIM-1 together with clusterin increased after 3 days of cisplatin exposure, whereas creatinine, BUN, and urinary total proteins increased after 5 days of cisplatin exposure. Similarly, in the drug-induced AKI rat models by Sasaki et al^34^, KIM-1 and clusterin levels were dramatically after administration of 100 mg/kg gentamicin for 7 days. Therefore, the release of endogenous renal Miox proteins may be faster than the inducible biomarkers KIM-1, clusterin, cystatin, and trefoil factor 3 as well as the traditional kidney injury indicators creatinine and BUN. These results suggested that Miox is a good biomarker for the early detection of drug-induced nephrotoxicity, since changes are detected at an early stage of proximal tubule cell injury.

Although many proteins are considered as potential biomarkers of kidney injury^16–19^, most of these biomarkers were less constant in blood or unstable in normal urine, and therefore rely more on other extra-renal pathologies. By contrast, our data exhibited that continued luminescence was detected in serum or urine stored at room temperature for 60 min. In addition, whether stored at 4 °C or − 20 °C for weeks, or repeatedly thawed and frozen, the luminescence in serum or urine was still detectable. This suggests that luminescence of Miox-NanoLuc has good stability in both serum and urine.

The next question was focused on the means by which highly expressed Miox-NanoLuc resulted in drug-induced proximal tubule cell injury. Previous studies demonstrated that necrosis/apoptosis and oxidant stress are the factors most at play in enhanced nephrotoxicity^35^. The Miox promoter includes an oxidant response element, and its overexpression will accentuate the generation of ROS in vitro^36,37^. Miox is upregulated during oxidative stress and hyperglycemia in vitro and in vivo, and this upregulation aggravates tubulointerstitial injury^38,39^. Under high glucose conditions, overexpression of Miox has been reported to cause mitochondrial dysfunctions in LLC-PK1 cells, including cytochrome C release, DNA fragmentation, and mitochondrial fission^37^. Moreover, in cisplatin-induced nephrotoxicity, the increased ROS generation in Miox-overexpressed cell could be related to pathology of the NAPDH oxidase system^37,40^. In AAI-induced nephrotoxicity, the effect was associated with a decrease in renal function, massive necrosis, increased inflammation, and oxidative stress^41^. Taken together, these results suggest that cisplatin- or AAI-induced nephrotoxicity appears to result in an increase in Miox-NanoLuc expression in *Miox-NanoLuc* transgenic mice, most likely via ROS generation.

Food and drug safety are vital to the health of populations. The first and most important food safety assessment is toxicity testing. Acute toxicity tests, based on the confirmation of half of the lethal dose (LD50) in experimental animals, indicating the degree of toxicity. After a repeated dose toxicity test, the animal will be sacrificed and observed for clinical pathology and histopathology to check for lesions; only then can the drug undergo efficacy tests and clinical trials. Despite these strict regulations, 2–3% of new drugs approved for marketing worldwide are later withdrawn due to toxicity problems undetected during the toxicity test. This situation results in not only serious damage to the health of users, but also huge losses to the pharmaceutical industry. It indicates that the original safety assessment method is still incomplete and urgently needs improvement. Therefore, our platform may be applied as a potential method to detect drug-induced nephrotoxicity before compounds are approved for use and marketed.

On the other hand, Miox levels in renal proximal tubular epithelium increased during hyperglycemia^36^. High-glucose ambience provides an environment conductive to the binding of binding of SP-1 to the Miox promoter and thereby regulate its expression^42^. Furthermore, it has been clinically found that diabetes, sepsis and ischemia/reperfusion increase sensitivity to AKI^43–45^. Miox expression significantly increased in patients with type 2 diabetes mellitus and correlated with tubulointerstitial lesiony^27^. Importantly, in diabetic patients without early signs of glomerular damage, serum and urine MIOX levels have increased. Thus, this platform can not only be directly applied to a wide range of drug/food testing, but also will be further extended to different disease models, such as diabetes, sepsis or ischemia/reperfusion.

In conclusion, we developed a new platform by which to quantify drug-induced nephrotoxicity by detecting the luminescence produced by the kidney-specific protein Miox-NanoLuc in mouse serum and urine. On this platform, luminescence produced by Miox-NanoLuc was increased at an early stage in the serum and urine of drug-treated transgenic mice before levels of serum BUN and creatinine increased and tubular cell injury could otherwise be noted. Moreover, this platform may be used for early detection of drug- and food-induced nephrotoxicity and as an animal model to investigate tubular cell injury.

## Supplementary information

Supplementary Information 1.

Supplementary Information 2.

Supplementary Information 3.
