# Supplementary material for: Kidney-based in vivo model for drug-induced nephrotoxicity testing
Source: Sci Rep. 2020 Aug 14;10:13640. doi: 10.1038/s41598-020-70502-3 (PMC7428004; doi:10.1038/s41598-020-70502-3)
Supplement: Supplementary file 1 — Supplementary Information 1. [file 41598_2020_70502_MOESM1_ESM.docx]

**Supplementary Figure 1. Expression profiles of Miox-NanoLuc in various organs of Miox-NanoLuc mice.** Expression of Miox-NanoLuc was determined in 30 organs of Miox-NanoLuc mice.

**Supplementary Figure 2. Pathological injury of kidney tissue in AAI-treated Miox-NanoLuc mice.** Miox-NanoLuc transgenic mice were treated 3.5 mg/kg AAI, and pathological injury of kidney were examined by H&E staining on day 3, 5, and 8. The kidney sections showed significant renal tubular cell cytolysis with detachment from the underlying basal lamina, loss of brush border and tubular cell vacuolization on day 5 and 8.
