# Supplementary figures and images for: Kidney-based in vivo model for drug-induced nephrotoxicity testing
Source: Sci Rep. 2020 Aug 14;10:13640. doi: 10.1038/s41598-020-70502-3 (PMC7428004; doi:10.1038/s41598-020-70502-3)

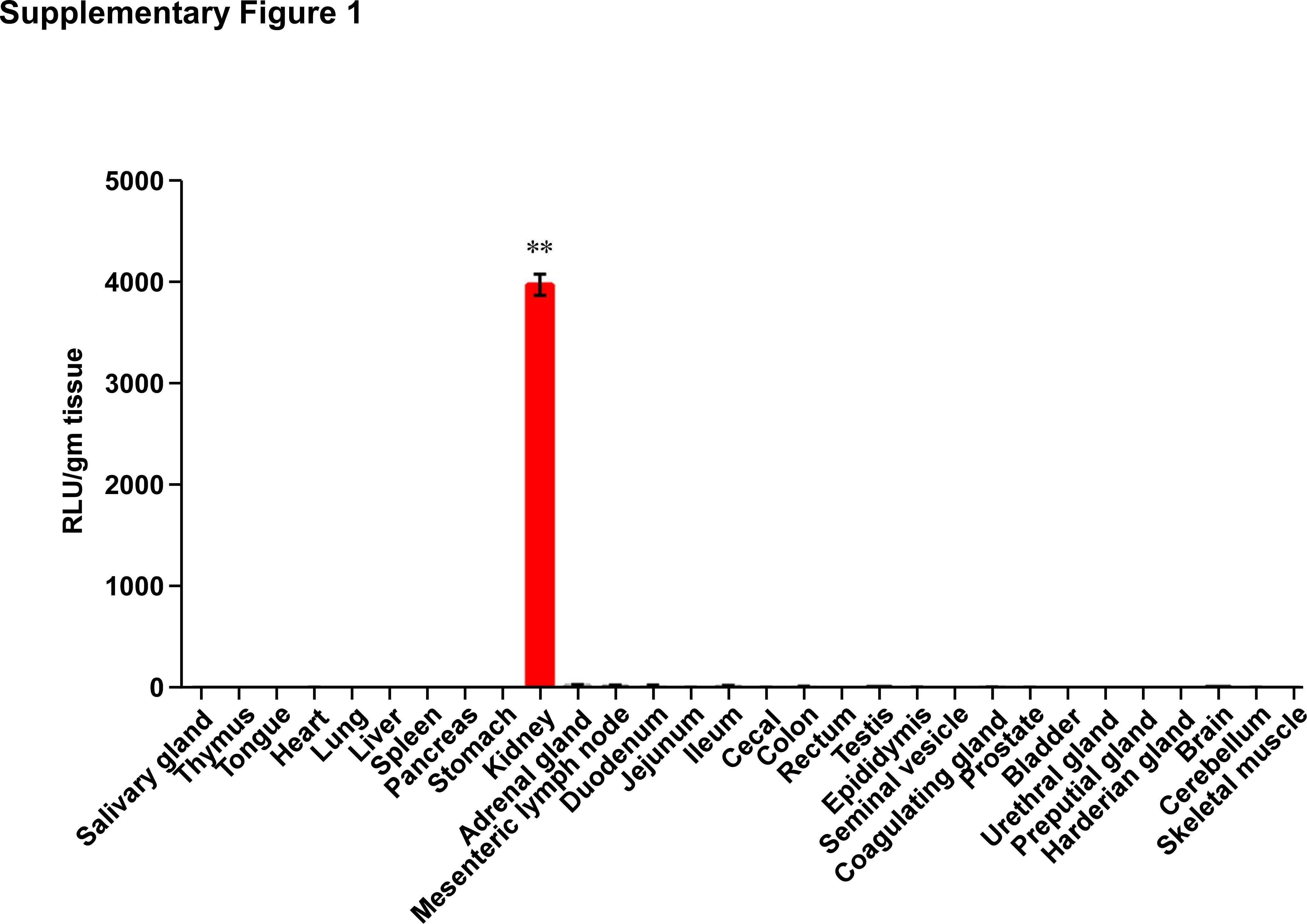

Supplement: Supplementary file 2 — Supplementary Information 2. [file 41598_2020_70502_MOESM2_ESM.jpg]

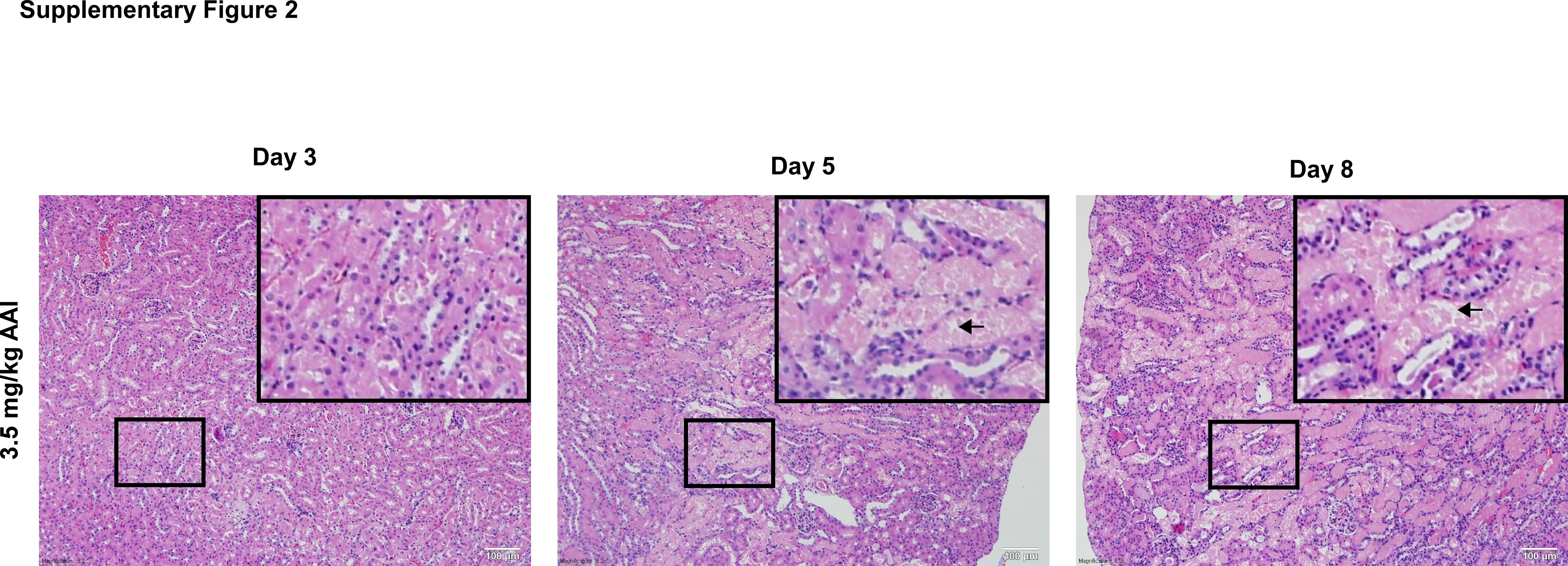

Supplement: Supplementary file 3 — Supplementary Information 3. [file 41598_2020_70502_MOESM3_ESM.jpg]
